# Prophylactic vertebroplasty procedure applied with a resorbable bone cement can decrease the fracture risk of sandwich vertebrae: long-term evaluation of clinical outcomes

**DOI:** 10.1093/rb/rbw037

**Published:** 2016-12-30

**Authors:** Pu Jia, Hai Tang, Hao Chen, Li Bao, Fei Feng, He Yang, Jinjun Li

**Affiliations:** 1 Department of Orthopaedics; 2 Department of Anesthesiology, Beijing Friendship Hospital, Capital Medical University, Beijing, China

**Keywords:** vertebroplasty, resorbable bone cement, sandwich vertebrae, spinal fracture

## Abstract

A sandwich vertebra is formed after multiple osteoporotic vertebral fractures treated by percutaneous vertebroplasty, which has a risk of developing new fractures. The purpose of our study was to (i) investigate the occurrence of new fractures in sandwich vertebra after cement augmentation procedures and to (ii) evaluate the clinical outcomes after prophylactic vertebral reinforcement applied with resorbable bone cement.

From June 2011 to 2014, we analysed 55 patients with at least one sandwich vertebrae and treated with percutaneous vertebroplasty. Eighteen patients were treated by prophylactic vertebroplasty with a resorbable bone cement to strengthen the sandwich vertebrae as the prevention group. The others were the non-prevention group. All patients were examined by spinal radiographs within 1 day, 6 months, 12 months, 24 months and thereafter.

The incidence of sandwich vertebra is 8.25% (55/667) in our study. Most sandwich vertebrae (69.01%, 49/71) are distributed in the thoracic–lumbar junction. There are 24 sandwich vertebrae (18 patients) and 47 sandwich vertebrae (37 patients) in either prevention group or non-prevention group, respectively. No significant difference is found between age, sex, body mass index, bone mineral density, cement disk leakage, sandwich vertebrae distribution or Cobb angle in the two groups. In the follow-up, 8 out of 37 (21.6%) patients (with eight sandwich vertebrae) developed new fractures in non-prevention’ group, whereas no new fractures were detected in the prevention group. Neither Cobb angle nor vertebral compression rate showed significant change in the prevention group during the follow-up. However, in the non-prevention group, we found that Cobb angle increased and vertebral height lost significantly (*P* < 0.05).

Prophylactic vertebroplasty procedure applied with resorbable bone cement could decrease the rate of new fractures of sandwich vertebrae.

## Introduction

Osteoporotic vertebral compression fractures (OVCFs) are a common complication of osteoporosis, which are also a major cause of morbidity and health costs among patients [1–3]. Percutaneous vertebroplasty (PVP) is a radiological procedure, which consists of percutaneous puncture and injection of polymethylmethacrylate (PMMA) into the broken vertebral body [4]. The PVP has been shown to be beneficial to patients in terms of pain relief and disability resolution [4–7]. However, there is much controversy that whether PVP could pose an increasing threat to the collapse of a vertebral body adjacent to the one previously treated with PMMA. Some studies [8, 9] have attempted to prove that PMMA injection would exaggerate force transmission to the adjacent vertebral bodies, developing new compression fractures. A sandwich vertebra is an intact vertebral body that is located between two vertebrae injected with cement [10]. It is hypothesized that the sandwich vertebrae is more likely to be broken, as a result from the strain concentration produced by the two adjacent vertebrae that sustained double load shift [8]. Until now, few studies concerning the fraction of sandwich vertebrae were reported. Moreover, there was no published data on the prevention of sandwich vertebral fractures until now.

Nowadays, the resorbable cement is widely applied in orthopedics, oral and dentofacial surgery and other fields [11–13]. This type of material has good osteoconductive properties and can act as conduction medium, which is usually composed of calcium sulfate, calcium phosphate and others. In our study, a type of resorbable calcium sulfate/calcium phosphate composite bone cement was injected into sandwich vertebra to prevent new fractures.

The aim of the study was to investigate the rates of new fractures in sandwich vertebrae. Moreover, we evaluated the prophylactic effect of the sandwich vertebrae reinforcement applied with the resorbable bone cement.

## Materials and methods

### Study population

Six hundred and sixty-seven patients with osteoporotic vertebral fractures who underwent PVP in our hospital from June 2011 to June 2014. The inclusion criteria were as follows: (i) older than 55 years old; (ii) multiple vertebral compression fractures with at least one sandwich vertebrae and (iii) patients who are able to understand the procedure and participate in the study. The exclusion criteria were (i) presence of a tumor, including multiple myeloma, vertebral hemangioma, vertebral metastasis and (ii) patients with secondary osteoporosis resulting from prior use of any glucocorticoid or other reasons. At last, 55 patients (10 males, 45 females; mean age 75.31 ± 7.91 years) were enrolled in the study. Eighteen patients agreed to the prophylactic vertebroplasty during the PVP, which are defined as the prevention group. The others are classified as non-prevention group. The mean follow-up was 18.5 ± 7.3 months (range, 9–36 months). All the patients were adequately informed prior to the study. All the investigations were approved by Ethics Committee of our hospital.

### Surgical procedures

All procedures have been performed fluoroscopically guided by unilateral pedicular approach under local anesthesia.

PVP procedure: local anesthesia (2% lidocaine and 1% rapivacaine, 1:1) was administered. Patients were positioned prone radiolucent table. The orientation of puncture was located in the anterior 3/4 of the vertebral body under the guidance of C-arm X-ray machine. Subsequently, 3–5 ml of PMMA (Osteopal V, Heraeus Medical, Germany) was injected until adequate filling of the vertebral body with lateral fluoroscopic guidance.

The prophylactic vertebroplasty: the protocol was the same with PVP and the two operations could be done at the same time. But, the cement injected was a resorbable calcium sulfate/calcium phosphate composite bone graft (GeneX, Biocomposites Ltd, UK).

### Evaluation of sandwich vertebrae

Postoperational examination including spinal radiographs was taken within 1 day, 6 months, 12 months, 24 months and thereafter. If a patient complaint a back pain or the spinal radiographs showed missing height of sandwich vertebral body, further magnetic resonance imaging or 99mTc bone scan was performed to confirm the presence of a new vertebral fracture.

### Data collection

**Figure 1:**
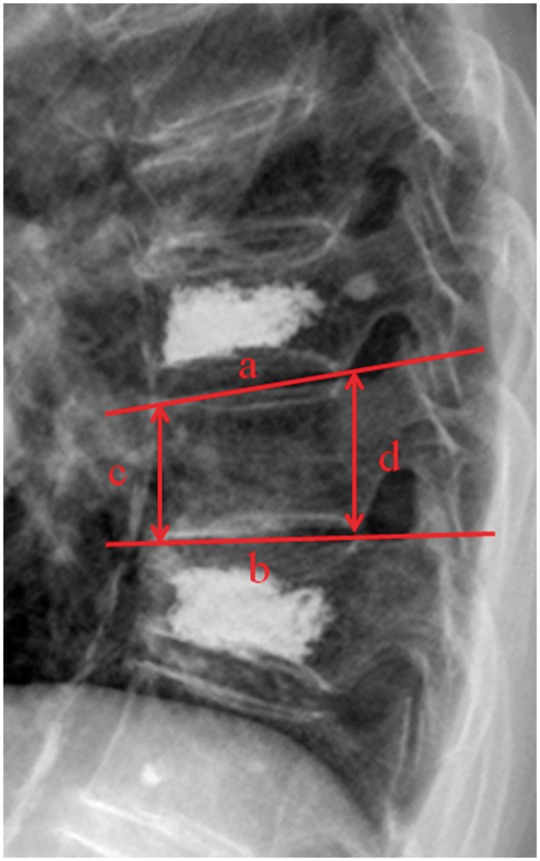
Lateral radiograph showed that the sandwich vertebral body located between two cement treated vertebrae. Cobb angle was the angle between **a** and **b**. The ratio between anterior height and posterior height was **c**/**d**

Data of patients within sandwich vertebra between two groups was analysed. A demographic database was created including age, sex, height, weight, bone mineral density (BMD) and follow-up period. The information was collected, including location of sandwich vertebra level, cement leakage into the intervertebral disk of sandwich vertebra. BMD of lumbar vertebrae (L1–4) and hip was measured by dual-energy X-ray absorptiometry (Hologic Inc., USA). The cobb angle of each sandwich vertebra between two groups was also measured. The height compression ratio of sandwich vertebra (anterior height/posterior height) was calculated to prevent errors caused by X-ray magnification (Fig. 1).

### Statistical analysis

All statistical analysis was conducted in SPSS 20.0. The age, body mass index (BMI), BMD and vertebral height compression rate were shown as average ± standard deviation. Between groups, independent sample T test was performed. Within the group, parried sample T test was performed. A chi square test was performed for data comparison. *P* < 0.05 was taken as statistically significant.

## Results

We performed PVP on 667 cases of osteoporotic vertebral fractures in the past 3 years. Fifty-five cases (10 males, 45 females; mean age 75.31 ± 7.91 years) with 71 sandwich vertebrae were involved in the study. The mean follow-up was 18.5 ± 7.3 months (range, 9–36 months). The incidence of sandwich vertebrae was 8.25% (55/667). The mean BMI was 22.66 ± 2.77 kg/m^2^. The BMD of lumbar and total hip was 0.688 ± 0.123 g/cm^2^ and 0.627 ± 0.226 g/cm^2^, respectively.

**Figure 2:**
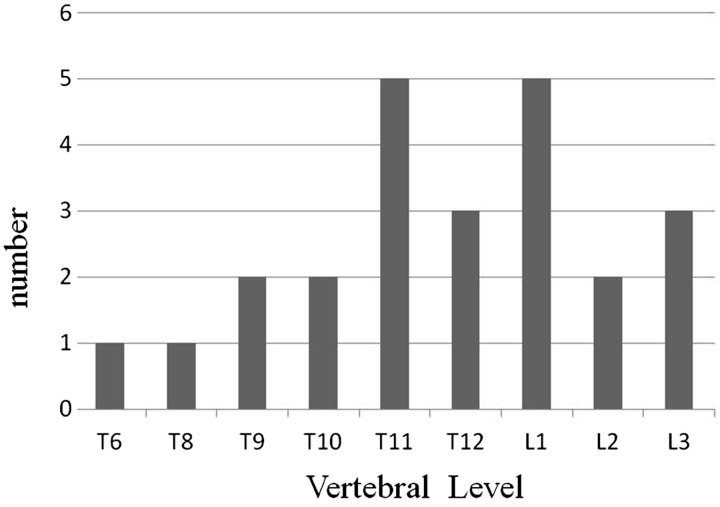
Locations of total 25 sandwich vertebrae in prevention group, whereas most of them were T11 and L1

**Figure 3:**
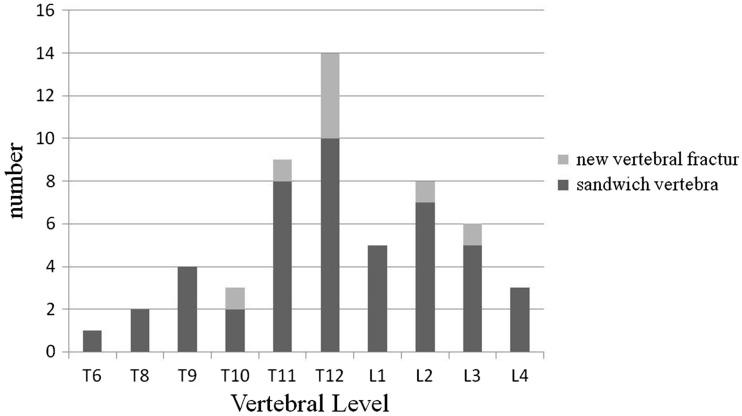
Locations of 47 sandwich vertebrae in non-prevention group and the eight sandwich vertebrae with new fractures

Overall, we observed 47 vertebrae (37 cases) in the non-prevention group and 24 vertebrae (18 cases) in the prevention group. There was no significant difference in age, sex, height, body weight, BMI or follow-up between the two groups (Table 1). In the non-prevention group, there were seven cases had cement leakage into the disc of the sandwich vertebra. However, in the prevention group, three patients suffered from cement leakage into the disc of the sandwich vertebra. No significant difference of occurrence rates of cement leakage had been observed (Table 1). Most of the sandwich vertebrae were located in the thoracic–lumbar junction (Figs 2 and 3). The distribution of sandwich vertebra was similar between the two groups (Table 1).

**Table 1:** Clinical data compared between two groups

	Prevention group	Non-prevention group	*P*-values
Patient, n	18	37	
Mean age, y	74.89 ± 6.18	75.51 ± 8.71	0.787
Sex, No. F:M	15:3	30:7	0.774
Mean height, m	1.60 ± 0.79	1.61 ± 0.83	0.703
Mean weight, kg	59.16 ± 7.92	58.19 ± 8.98	0.695
Mean BMI, kg/m^2^	23.03 ± 2.10	22.46 ± 3.08	0.471
L1–4 mean BMD	0.678 ± 0.118	0.691 ± 0.127	0.769
Hip total BMD	0.601 ± 0.120	0.641 ± 0.269	0.604
Cement disk leakage, n	3	8	0.666
Sandwich vertebra, n	24	47	
Thoracic–lumbar, n	17	32	0.813
Cobb angle of SV	5.28 ± 3.62	3.92 ± 3.17	0.195
Compression rate of SV	0.90 ± 0.10	0.93 ± 0.07	0.107

No significance between two groups. BMI, body mass index; BMD, bone mineral density; SV, sandwich vertebra. Cobb angle and compression rate of sandwich vertebra were measured by lateral spinal radiographs within 1 day after procedure.

**Figure 4:**
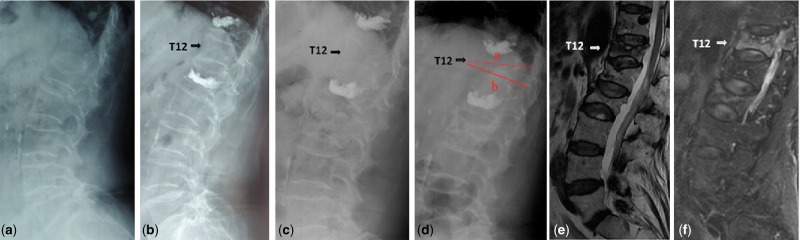
Images obtained in a 74-year-old woman with OVCFs. (**a**) The lumbar lateral radiograph before procedure. (**b**) T12 is a sandwich vertebral body located between L1 and T11 preciously treated with vertebral cement augmentation. (**c**) Follow-up after 12 months showed no loss of T12 height. (**d**) Patients felt back pain after 27 months after procedure. Lateral radiograph showed obvious compression within T12. (**e**) T2-weighted image indicated high signal intensity within T12. (**f**) Image showed high signal intensity within T12, indicating fresh vertebral fractures

**Figure 5:**
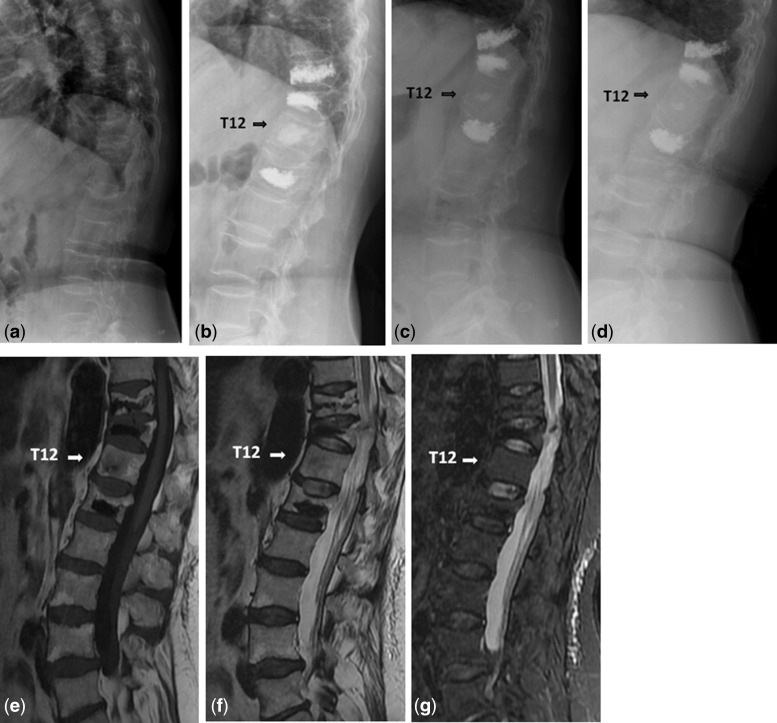
Images obtained in a 63-year-old woman with OVCFs. (**a**) The lumbar lateral radiograph before procedure. (**b**) T10, T11 and L2 were treated with vertebral cement augmentation, where T12 was a sandwich vertebra. (**c**) Follow-up after 12 months showed no loss of T12 height. (**d**) Follow-up after 22 months showed no loss of T12 height. (**e** and **f**) T1- and T2-weighted images indicated low intense signal, indicating the calcium phosphate component which remains intact for years providing an osteoconductive matrix for new bone ingrowth. (**g**) Image showed no high signal intensity

In the non-prevention group, eight patients (21.6%) had experienced eight sandwich vertebrae (17%) fractures. We described pre- and postoperation images of a case in the non-prevention group (Fig. 4). However, there was no patients who suffered new fracture of sandwich vertebra in the prevention group. We also presented pre- and postoperation images of a prophylactic case (Fig. 5). Significant difference of the new fracture occurrence was observed between the two groups (Table 2). However, rates of new fracture of sandwich vertebra were 21.9% in thoracic–lumbar junction (T11-L1 segment), compared with 6.7% in the other segments (*P* = 0.381, Table 3). Furthermore, we showed that the cobb angle increased and vertebral anterior height decreased significantly in the non-prevention group (*P* < 0.05, Table 4). No significant change of cobb angle or vertebral height was observed in the prevention group.

**Table 2:** **** Incidence of new fractures in the two groups

	Patient, n			Vertebra, n		
	New F	No F	(%)	New F	No F	(%)
Prevention group	0	18	0	0	24	0
Non-prevention group	8	29	21.6	8	39	17.0
Chi square test	*P* = 0.033*			*P* = 0.032*		

New F, new fracture; No F, no fracture. The difference is statistically significant *P < 0.05.

**Table 3:** Decrease of new fractures in non-prevention group

	Vertebra, n		
	New F	No F	(%)
Thoracic–lumbar junction	7	25	21.9
Non-thoracic–lumbar junction	1	14	6.7
Chi square test	*P* = 0.381		

New F, new fracture; No F, no fracture. The difference is no statistically significant.

**Table 4:** Cobb angle and vertebral compression ratio between two groups

	Cobb angle			Vertebral compression ratio		
	Post op	L-FLU	*P*	Post op	L-FLU	*P*
Prevention group	5.28 ± 3.62	5.85 ± 3.91	0.285	0.90 ± 0.10	0.88 ± 0.09	0.089
Non-prevention group	3.92 ± 3.17	5.87 ± 4.27	0.000*	0.93 ± 0.07	0.89 ± 0.12	0.002*

L-FLU, last follow-up. In the non-prevention group, the Cobb angle increased and the vertebral compression rate decreased at follow-up (*P < 0.05).

## Discussion

PVP is gaining popularity in the treatment of OVCFs, for its minimal invasion and rapid pain relief [14–16]. However, the vertebral bodies adjacent to the vertebrae previously treated by PMMA are more likely to develop new fractures. Most studies showed that the rates of new fractures in adjacent vertebra were relatively higher than non-adjacent level [10, 17–20]. In this study, we found that the rates of new fractures in sandwich vertebral fractures were 21.60%, which was similar with the previous report (21.43%) [10]. It seemed that the sandwich vertebrae were easier to fracture than the adjacent vertebral fractures.

It was altered biomechanics in the treated vertebrae that developed an adjacent segment fracture [8, 9]. But, there were no reports about altered biomechanics in sandwich vertebrae. It is generally recognized that sandwich vertebra sustained double load shift and suffered strain concentration produced by the two adjacent augmented vertebrae. The risk factors of new fractures of adjacent vertebra were investigated in many studies. The amount of PMMA injected per vertebral body [8], intradiskal cement leakage [21–24], location of the adjacent vertebra [25], local kyphotic angulation [10] and lower BMD [17, 24] were all proved to increase risk of developing new fractures in adjacent vertebra. However, no significant difference of the risk factors was observed between the two groups. Interestingly, most of the sandwich vertebrae in our study were located in the thoracic–lumbar junction, especially T11-L1 segment. But, there was no statistically significant difference. The sample size may be not enough or others reasons.

Prophylactic vertebral reinforcement was limited *in* *vitro* studies so far. The injected PMMA was shown to increase the loading of on-axis [26, 27] or off-axis [28]. Regardless of beneficial results in the research, the spinal column was far more complicated than the single sample. There were many limits for PMMA in prophylactic vertebral reinforcement, for its biomechanical properties such as too high stiffness, strength and inabsorable. So it remained controversial in prophylactic vertebral reinforcement with PMMA [29, 30]. In Uebelhart’s report [31], they found refractures in the prophylactic vertebral reinforcement with PMMA. The reasons might be the increase of bone fragility caused by long history of glucocorticoid intake. Meanwhile, it was confirmed that only the endplate-to-endplate filling of PMMA would increase the vertebral intensity [32, 33]. It seemed that sandwich vertebrae was more easily to refractrure when the PMMA was not adequately filled, especially in patients with increased bone fragility. Moreover, during PMMA polymerization, the induced heat and MMA toxicity damage has been proved in some studies [34, 35], resulting in aseptic inflammation and bone necrosis, thereby developing new fractures [36].

Nowadays, some absorbable bone graft substitutes were studied *in vitro* and *in vivo* trials [11, 12, 37–40], including calcium sulfate, calcium phosphate and the mixture of both materials. In our research, the bioactive and resorbable cement was GeneX, which was an injectable bone substitute consisting of calcium sulfate and beta-tricalcium phosphate with a weight ratio of 1/1. The calcium sulfate/calcium phosphate composite bone cement powder had a reaction with curing liquid with 10–15 min of coagulation, which was similar to PMMA. Once mixed, powder and liquid composed a viscous paste able to be easily injected. During the complete hardening period, the composite bone cement became solid providing mechanical strength. The calcium sulfate will be gradually resorbed allowing the implant to be remodeled through bone ingrowth [37]. However, the strength of calcium sulfate alone is too weak, and its rate of resorption is too high. The calcium phosphate could slow down the absorption speed of calcium sulfate and at the same time act as an osteoconductive template for new bone ingrowth. The calcium phosphate could embedded inside new bone tissue during the new bone ingrowth [11, 12, 38–40]. However, there were few reports about prophylactic vertebral reinforcement and the localized treatment of osteoporosis applied with resorbable composite bone cement. In our opinion, the prophylactic vertebroplasty with the resorbable composite bone cement is not only used for the prevention of the new fracture but also a novel localized treatment for osteoporosis. Because the resorbable calcium sulfate/calcium phosphate composite bone cement could help the induction of new bone and increase the bone strength in patients with osteoporosis. As the strength of sandwich vertebra, or others with a risk of fractures, could be increased by the calcium sulfate/calcium phosphate composite bone cement, the possibility of the new fracture would decrease.

There were some limitations in our study. First of all, the level of osteoporosis was one of the risk factors of developing new fractures. Our study didn’t involve the effect of antiosteoporosis drugs on new fractures. Besides, the scale of the group was relatively small, especially for the prevention group. Therefore, we needed to expand the size of sample to further investigate the risk factors of new sandwich vertebral fractures. Finally, we couldn’t get histological sections of the sandwich vertebral during the follow-up, which can confirm osteogenesis effect of the absorbable bone graft.

## Conclusion

PVP, a safe and effective procedure, is used to treat multiple thoracic or/and lumbar fractures caused by osteoporosis, which could form the sandwich vertebra. It has a risk of developing new fractures in the sandwich vertebra. However, prophylactic vertebroplasty applied with calcium sulfate/calcium phosphate composite bone cement can decrease the rate of new fractures in sandwich vertebra.

*Conflict of interest statement*. None declared.
